# The use of chest ultrasonography in suspected cases of COVID-19 in the emergency department

**DOI:** 10.2144/fsoa-2020-0127

**Published:** 2020-11-30

**Authors:** Enrico Allegorico, Carlo Buonerba, Giorgio Bosso, Antonio Pagano, Giovanni Porta, Claudia Serra, Pasquale Dolce, Valentina Minerva, Ferdinando Dello Vicario, Concetta Altruda, Paola Arbo, Teresa Russo, Chiara De Sio, Nicoletta Franco, Gianluca Ruffa, Cinzia Mormile, Francesca Cannavacciuolo, Valentina Mercurio, Gelsomina Gervasio, Giuseppe Di Costanzo, Alfonso Ragozzino, Luca Scafuri, Gaetano Facchini, Fabio Numis

**Affiliations:** 1 Department of Emergency Medicine “Santa Maria delle Grazie” Hospital, 80078 Pozzuoli, Italy; 2 Department of Oncology & Hematology, Regional Reference Center for Rare Tumors, AOU Federico II of Naples, 80131 Naples, Italy; 3 Centro di Referenza Nazionale per l’Analisi e Studio di Correlazione tra Ambiente, Animale e Uomo, Istituto Zooprofilattico Sperimentale del Mezzogiorno, 80055, Portici (Na), Italy; 4 A.O.R.N.“San Giuseppe Moscati”, Internal Medicine Department, 83100 Avellino, Italy; 5 Department of Public Health, Federico II University, 80131 Naples, Italy; 6 Department of Diagnostic Imaging “Santa Maria delle Grazie” Hospital, 80078 Pozzuoli, Italy; 7 Department of Medical & Surgical Sciences, Università della Campania “Luigi Vanvitelli”, 80131 Naples, Italy; 8 Internal Medicine, AOU Federico II of Naples, 80131 Naples, Italy; 9 Department of Hospital Medicine, Unit of Medical Oncology, ASL Napoli 2 Nord, “S.M. delle Grazie” Hospital, Pozzuoli (NA), Italy; 10 Department of Clinical Medicine & Surgery, University Federico II of Naples, Via Pansini 5, 80131 Naples, Italy

**Keywords:** COVID-19, lung ultrasound, RT-PCR, SARS-CoV-2

## Abstract

**Aim:**

Severe acute respiratory syndrome coronavirus 2 (SARS-CoV-2) virus-specific reverse transcriptase-polymerase chain reaction (RT-PCR) represents the diagnostic gold standard. We explored the value of chest ultrasonography to predict positivity to SARS-CoV-2 on RT-PCR in suspected COVID-19 cases.

**Patients & methods:**

Consecutive patients with suspect COVID-19 were included if they had fever and/or history of cough and/or dyspnea. Lung ultrasound score (LUSS) was computed according to published methods.

**Results:**

A total of 76 patients were included. A 3-variable model based on aspartate transaminase (AST) > upper limit of normal, LUSS >12 and body temperature >37.5°C yielded an overall accuracy of 91%.

**Conclusion:**

A simple LUSS-based model may represent a powerful tool for initial assessment in suspected cases of COVID-19.

## Lay abstract

The gold standard for diagnosis of COVID-19 is RT-PCR. During a pandemic emergency, it may be useful to identify suspect symptomatic patients who may safely be observed without undergoing testing for COVID-19. In this work, a simple model based on the findings of lung ultrasound, AST levels and fever showed an overall accuracy of 91% to predict the results of RT-PCR.

With approximately 233,000 confirmed cases and 33,000 deaths in Italy alone and approximately 6 million cases and 370,000 deaths worldwide as of 1 June 2020, COVID-19, the disease caused by the ssRNA beta-coronavirus SARS-CoV-2 [1], has represented a public health emergency for the past 6 months and has posed major diagnostic and therapeutic challenges, especially in patients with comorbid conditions such as cancer [2]. The wide spectrum of severity of the clinical presentation of COVID-19 encompasses asymptomatic/mild disease, reported in approximately 80% of patients; severe disease with lung infiltrates, hypoxia, dyspnoea in approximately 15% of patients; and deadly disease with multiorgan dysfunction, respiratory failure and shock reported in approximately 5% of cases [3]. COVID-19 patients who require intensive care and mechanical ventilation have a grim prognosis, with no pharmacological options with an established effect on survival [4] and a mortality rate as high as 60% [5].

Besides therapeutic difficulties, COVID-19 may also present diagnostic hurdles, as the gold standard for diagnosis is SARS-CoV-2 virus-specific reverse transcriptase-polymerase chain reaction (RT-PCR), with even higher sensitivity provided by droplet digital polymerase chain reaction (PCR) [6]. Unfortunately, RT-PCR testing has multiple disadvantages, including its relative cost (dozens of euros per test), its processing time (up to 2 days) and the worldwide shortage of test kits [7].

As serologic tests do not represent good alternatives for diagnosis of suspect cases in the emergency setting, with sensitivities of commercially available ELISA and rapid diagnostic tests below 40% within the first 5 days from onset of the disease [8], models based on clinical, radiological and laboratory findings that can safely reduce the need for PCR-RT testing in selected suspect patients are of utmost clinical importance.

Computed tomography (CT) scans analyzed with the aid of artificial intelligence [9] as well as clinical models based on medical and personal history, radiological, clinical and laboratory findings [10] have shown high accuracy in large retrospective studies. In this setting, lung ultrasound may also play a role. Lung ultrasound has an established diagnostic value in patients with acute respiratory failure, with the additional advantage of being performed bedside and avoiding the patient’s transferal to radiology [11]. During the COVID-19 pandemic, several authors have proposed its usefulness for early diagnosis of suspect cases [12], although limited data are available in this setting.

In this retrospective study, we aimed to explore the value of lung ultrasonography to predict RT-PCR test results on admission to the emergency department. The predictive value of CT findings and of commonly available clinical and biochemistry variables was also explored.

## Materials & methods

### Study design & setting

This retrospective study was carried out at the Emergency Department of the Santa Maria delle Grazie Hospital (Naples, Italy) from 1 March to 30 April 2020. All patients included in the study were followed-up from admission to the emergency department (Day 1) for 7 days. Consecutive patients were included if they had either fever (body temperature >37.5°C measured using infrared thermometer) and/or history of cough and/or dyspnea within the previous 48 h as assessed on Day 1. RT-PCR nasal swab test had to be performed on Day 1. Testing was repeated on Days 2–7 on an individual case basis according to the physician’s judgement. On Day 1, patients were also required to have undergone a complete routine biochemistry, arterial blood gas test, physical examination and thoracic ultrasound, while a thoracic CT scan was to be performed on Days 1–3. Patients with any missing clinical, biochemistry and radiological data (thoracic ultrasound, CT scan) were excluded from the study. The end point of the study was RT-PCR test result. Patients positive for SARS-CoV-2 on Day 1 or on retesting on Days 2–7 (if performed) were considered as positive, while patients who tested negative for SARS-CoV-2 on Day 1 and on subsequent testing on Days 2–7 (if performed) were considered negative. The objective of this study was to construct a model based on thoracic ultrasound findings to predict nasal swab RT-PCR test result

### Data collection

The following data, obtained by reviewing patients’ clinical charts, were collected in an Excel data-sheet in an anonymized form: demographic characteristics (age, sex), vital signs (respiratory rate, temperature), history of cough and/or dyspnea within the past 48 h, blood biochemistry tests (lactate dehydrogenase (LDH), creatine kinase, C-reactive protein, procalcitonin, D-dimer, creatinine, fibrinogen, prothrombin time (PT), activated partial thromboplastin time (aPTT), AST, alanine transaminase (ALT), white blood cell count, neutrophil count, platelet count), lung ultrasound findings (Lung UltraSound Score [LUSS]) and chest CT scan findings (CT severity index, CO-RADS score). Chronic Kidney Disease Epidemiology Collaboration and APACHE II (Acute Physiology and Chronic Health Evaluation II) were computed according to published methods [13,14]. LUSS [15], CO-RADS score and CT severity index [16] were obtained according to published methods by experienced radiologists. Briefly, LUSS was computed assessing 12 lung regions, which were graded between 0 and 3 according to the degree of aeration loss, so the score can vary between 0 and 36. Conversely, the level of suspicion was graded from very low or CO-RADS 1 up to very high or CO-RADS 5 on CT scan findings. The CT severity score was computed by estimating the degree of parenchymal opacification in 20 lung regions on a scale from 0 to 2, so the overall score can vary between 0 and 40. SARS-CoV-2 tests were performed in one of the laboratories of the CORONET Network of the Campania Region following the WHO protocol. Two RNA-dependent RNA polymerase targets were used for RT-PCR (Ct <40). Details of the protocol used for RT-PCR are available online [17].

### Statistical analysis

The frequencies of categorical variables were reported, while median (interquartile range) was used for description of continuous variables. Univariate analysis to assess predictors of positive RT-PCR test was performed by using simple logistic regression analysis. Continuous variables were dichotomized using their median value. Multivariate analysis was performed including all variables that were significantly associated with a positive RT-PCR test at univariate analysis except for those that showed multicollinearity issues. Finally, all variables with a p-value < 0.05 at multivariate analysis were included in the final model. Model accuracy was assessed by using receiver operating characteristic curves and by computing the area under the curve (AUC). All tests were two-sided. The R 3.6.0 software environment for statistical computing was used for all statistical analyses.

## Results

**Figure 1.**
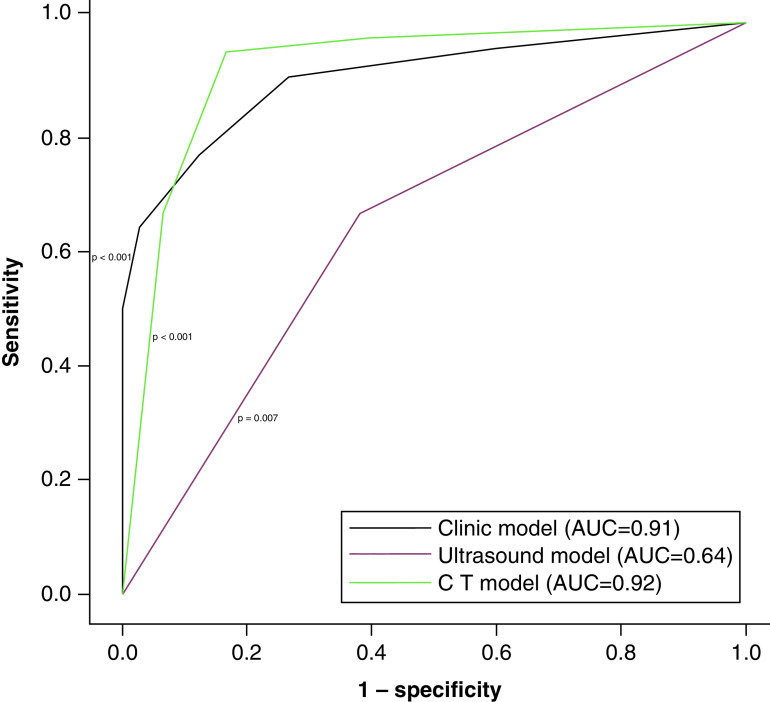
Receiver operating characteristic curves of the models.

A total of 76 patients who had been admitted to the Emergency Department of the Santa Maria delle Grazie Hospital from 1 March to 30 April 2020 were included in this retrospective analysis. All patients had fever (body temperature >37.5°C measured using infrared thermometer) and/or history of cough and/or dyspnea within the previous 48 hours. Approximately two thirds were men, while median age was 68.5 years (interquartile range, 52.7; 78.2). Characteristics of the study population are detailed in Table 1. Overall, a total of 42 patients (55.2%) were positive for SARS-CoV-2 on RT-PCR testing. Four patients were negative on Day 1 but tested positive on retesting on days 2–7 and were considered positive. A total of 34 patients were negative on Day 1. Of these, seven patients were retested on Days 2–7 and confirmed to be negative. At univariate analysis, LDH, C Reactive Protein, procalcitonin, creatinin, Chronic Kidney Disease Epidemiology Collaboration, AST, white blood cells, LUSS, body temperature were all significantly associated with RT-PCT test outcomes (Table 2). At multivariate analysis, only AST (odds ratio [OR] = 5.78, p = 0.028), body temperature (OR = 46.0, p < 0.001) and LUSS (OR = 8.27, p = 0.021) were significantly associated with RT-PCR test results (Table 3). These three variables were used to construct a clinical model to predict RT-PCR test outcomes. In order to obtain a generalizable model, we used upper limit of normal (i.e., 34 UI/l) and the temperature of 37.5°C as thresholds to dichotomize AST levels and body temperature, respectively, while LUSS was dichotomized considering the median value (i.e., 12). In this 3-variable model, AST > upper limit of normal, LUSS >12 and body temperature >37.5°C were respectively associated with an OR = 5.29 (p = 0.021), an OR = 10.2 (p = 0.006) and an OR = 49.2 (p < 0.001), with an overall accuracy of the model of 91%. When CT findings were analyzed on bivariate analysis, we found that CO-RADS score >3 versus <=3 was associated with an OR = 77.2 (p = <0.001) and a CT Severity index >= median versus < median was associated with an OR = 3.22 (p = 0.151) . The AUC of the three-variable clinical model based on LUSS, AST and fever (AUC = 91%) compared favorably with the AUC of LUSS alone (AUC = 64%) and was numerically similar to the AUC of CT-based model (AUC = 92%) (Figure 1).

**Table 1.** Characteristics of the entire cohort.

Variable		Positive for SARS-CoV-2	Negative for SARS-CoV-2
Absolute number (%)			
Male gender	52 (68.4%)	29 (69%)	23 (67.6%)
Cough	35 (46.1%)	23 (54.8%)	12 (35.3%)
Dyspnea	57 (75%)	31 (73.8%)	26 (76.5%)
Median (interquartile range)			
Age (years)	68.5 (52.7; 78.2)	68.5 (51; 80)	67.5 (52.7; 78.2)
LDH (IU/l)	287 (226; 430)	366 (251; 570)	258 (211; 341)
Creatinine kinasis (IU/l)	101.5 (45.7; 225)	98 (38; 224)	101.5 (58.7; 242.5)
C reactive protein (mg/dl)	5.1 (1.34; 15.4)	10.7 (3.7; 21.2)	1.59 (0.67; 6.61)
Procalcitonin (ng/ml)	0.07 (0.03; 0.22)	0.12 (0.05; 0.46)	0.04 (0.02; 0.1)
D-Dimer (ng/ml)	514 (288; 2043)	539 (288; 2191)	493 (249; 2074)
APACHE II score	11 (7; 15)	11 (6; 15)	10.5 (8.7; 16)
CKD-EPI (ml/min per 1.73 m^2^)	78 (50.7; 94)	70 (50.7; 84))	85.5 (48.7; 101)
Fibrinogen (mg/dl)	395 (277; 544)	449 (281; 562)	333 (269; 544)
PT (s)	75 (65; 87)	74 (65; 81)	78 (65.7; 95.2)
APTTs (s)	33.3 (30.6; 40)	32.6 (31.2; 40)	34.2 (30.1; 41.1)
AST (IU/L)	32 (23; 60)	44 (28; 65.5)	26 (18.5; 47)
ALT (IU/L)	24 (15; 50)	28 (18; 51)	22 (12; 44)
White blood cell (/μl)	9750 (7500; 13,525)	8300 (5300; 12,425)	10,950 (8600; 15,100)
Neutrophils (/μl)	7750 (5450; 10,900)	6250 (3525; 10,250)	9100 (6875; 11,600)
Lymphocytes (/μl)	1200 (775; 1800)	1200 (775; 1558)	1250 (700; 2250)
Monocytes (/μl)	600 (475; 812)	600 (400; 762)	700 (500; 1100)
Platelets (×10^3^/μl)	219 (160; 321)	199 (146; 321)	241 (194; 342)
LUSS	12 (4; 16.5)	14 (10; 20)	8 (0; 12)
Ratio of partial pressure arterial oxygen/fraction of inspired oxygen (mmHg)	295 (235; 385)	295 (218; 388)	297 (255; 386)
Respiratory rate (per min)	21 (18; 25)	22 (18; 25)	20 (18; 24)
Body temperature (C°)	37.5 (36; 38)	37.8 (37.5; 38.1)	36 (36; 37)

Data are reported as number of patients (%) or median (IQR), as appropriate. p-values are obtained using simple logistic regression analysis, dichotomizing each quantitative explanatory variable about its median value.

ALT: Alanine transaminase; APACHEII: Acute Physiology and Chronic Health Evaluation II; APTT: activated partial thromboplastin time; AST: Aspartate transaminase; CKD-EPI: Chronic Kidney Disease Epidemiology Collaboration; IQR: Interquartile range; LDH: Lactate dehydrogenase; LUSS: Lung ultrasound score; PT: Prothrombin time.

**Table 2.** Univariate analysis of predictors of positive RT-PCR test for SARS-CoV-2.

Variable	Analyzed as	OR (95% CI)	p-value
Male gender	Male vs female	1.07(0.40–2.83)	0.896
Cough	Presence vs absence	2.22 (0.89–5.74)	0.093
Dyspnea	Presence vs absence	0.87 (0.30–2.46)	0.790
Age (years)	≥median vs <median	1 (0.40–2.48)	0.999
LDH (IU/l)	≥median vs <median	2.98 (1.18–7.82)	**0.023**
Creatinine kinasis (IU/l)	≥median vs <median	1 (0.40–2.48)	0.999
C reactive protein (mg/dl)	≥median vs <median	4.8 (1.85–13.2)	**0.002**
Procalcitonin (ng/ml)	≥median vs <median	4.58 (1.78–12.5)	**0.002**
D-Dimer (ng/ml)	≥median vs <median	1.24 (0.50–3.09)	0.645
APACHE II score	≥median vs <median	1.10 (0.44–2.73)	0.836
CKD-EPI (ml/min per 1.73 m^2^)	≥median vs <median	0.37 (0.14–0.93)	**0.038**
Fibrinogen (mg/dl)	≥median vs <median	1.53 (0.62–3.85)	0.357
PT (s)	≥median vs <median	0.68 (0.27–1.70)	0.408
APTTs (s)	≥median vs <median	0.68 (0.27–1.70)	0.408
AST (IU/l)	≥median vs <median	3.54 (1.39–9.45)	**0.009**
ALT (IU/l)	≥median vs <median	1.44 (0.58–3.62)	0.436
White blood cell (/μl)	≥median vs <median	0.34 (0.13–0.85)	**0.023**
Neutrophils (/μl)	≥median vs <median	0.42 (0.16–1.05)	0.067
Lymphocytes (/μl)	≥median vs <median	1.47 (0.59–3.70)	0.407
Monocytes (/μl)	≥median vs <median	0.53 (0.20–1.33)	0.180
Platelets (×10^3^/μl)	≥median vs <median	0.42 (0.16–1.05)	0.067
LUSS	≥median vs <median	3.23 (1.28–8.51)	**0.015**
Ratio of partial pressure arterial oxygen/fraction of inspired oxygen (mmHg)	≥median vs <median	1 (0.40–2.48)	0.999
Respiratory rate (per min)	≥median vs <median	1.90 (0.77–4.84)	0.168
Body temperature (C°)	≥median vs <median	17 (5.76–58)	**<0.001**

Data are reported as number of patients (%) or median (IQR), as appropriate. p-values are obtained using simple logistic regression analysis, dichotomizing each quantitative explanatory variable about its median value.

ALT: alanine transaminase; APACHEII: Acute Physiology and Chronic Health Evaluation II; APTT: activated partial thromboplastin time; AST: Aspartate transaminase; CKD-EPI: Chronic Kidney Disease Epidemiology Collaboration; IQR: Interquartile range; LDH: Lactate dehydrogenase; LUSS: Lung ultrasound score; OR; Odds ratio; PT: Prothrombin time.

**Table 3.** Multivariate analysis of predictors of positive RT-PCR test for SARS-CoV-2.

Variable	Analyzed as	OR (95% CI)	p-value
CKD-EPI	≥median vs <median	0.59 (0.13–2.52)	0.472
AST	≥median vs <median	5.98 (1.41–33.4)	**0.023**
White blood cell count	≥median vs <median	0.28 (0.06–1.13)	0.081
LUSS	≥median vs <median	6.97 (1.52–51.1)	**0.024**
Body temperature (°C)	≥median vs <median	51.4 (10.6–442)	**<0.001**

Data are reported as number of patients (%) or median (IQR), as appropriate. p-values are obtained using simple logistic regression analysis, dichotomizing each quantitative explanatory variable about its median value.

AST: Aspartate transaminase; CKD-EPI: Chronic Kidney Disease Epidemiology Collaboration; IQR: Interquartile range; LUSS: Lung ultrasound score; OR: Odds ratio.

## Discussion

Over the past decade, the use of thoracic ultrasound has become more and more widespread in clinical practice, especially in the emergency setting, where it represents a readily available, powerful diagnostic tool that provides valuable clinical information without the need to transfer the patient to radiology and without the use of ionizing radiations [11]. Besides representing a reliable tool for the diagnosis of pneumonia [18], thorax ultrasound can be particularly useful to monitor patients with acute respiratory distress syndrome [19], as it can capture changes in regional lung aeration associated with position [20], fluid loading [21] as well as positive end-expiratory pressure [22].

Despite the paucity of supporting experimental data, several experts have suggested that thorax ultrasound can be used to assess severity of pulmonary involvement in COVID-19 patients, with clear advantages in terms of reduced use of CT and x-ray (RX) scans as well as reduced risk of exposure to the virus for healthcare workers [12,23,24]. Kalafat *et al.* reported the case of a woman with a negative RT-PCR result, who was retested after a positive lung ultrasound and correctly diagnosed with COVID-19 [25]. In a retrospective series including ten COVID-19 pediatric patients, thorax ultrasound allowed detection of signs of lung involvement, including vertical artifacts (70% of cases), pleural abnormalities (60% of cases), areas of white lung (10% of cases) and subpleural consolidations (10% of cases) [26]. In another retrospective study by Lu *et al.* [27] including 30 COVID-19 patients assessed by the use of thorax ultrasound and CT, thorax ultrasound yielded a diagnostic accuracy compared with CT of 76.7, 76.7 and 93.3%, in patients with mild, moderate and severe lung involvement respectively. Finally, in a series of 100 patients (31 positive for SARS-CoV-2 on RT-PCR) thorax ultrasound was associated with an overall accuracy of 82%, with a sensitivity of 97% (83–100%), a specificity of 62% (50–74%), a positive predictive value of 54% (41–98%) and negative predictive value of 98% (88–99%) [28].

We are the first to compute LUSS in a series of consecutive patients admitted to the Emergency Department with clinical suspicion of COVID-19 based on fever (t°C >37.5) and/or history of cough and/or dyspnea within the previous 48 h. Our main finding was that LUSS was significantly associated with positivity to SARS-CoV-2 on RT-PCR at univariate analysis. After adjusting for multiple clinical and laboratory findings, LUSS was associated with positive RT-PCR with an OR of 8.27 and AUC of 0.64. Multivariate analysis showed that a simple model including LUSS, abnormal AST (>34 UI/l) and fever (body temperature >37.5°C) yielded an overall accuracy of 91%. In this regard, it is interesting to note that elevated AST levels have been associated with adverse outcomes in COVID-19 patients [29]. Furthermore, we found comparable accuracy of our proposed ultrasound-based model and CT-based model (AUC = 91 vs 92%).

Our study suffers from multiple limitations. First, we could include only a limited sample size of patients admitted to the Emergency Department setting, due to the favorable course of the pandemic at a local level, with fortunately only a few new daily cases since last May in the Campania region. Second, our study has a retrospective design and the data collected have been obtained in the setting of clinical practice rather than within an experimental trial. Third, not all patients with an initial negative PCR test were retested, so some falsely negative patients may have been missed.

## Conclusion

A simple model based on computation of LUSS, evaluation of abnormal AST findings and fever may represent a powerful tool for initial assessment in suspect cases of COVID-19. Patients at low risk may be safely admitted to the hospital without the need of being isolated in the same area of patients at high risk for COVID-19. The use of our model may allow to save both CT scans and RT-PCR testing. Prospective validation is warranted.

## Future perspective

Results from this study should be first confirmed in a larger series (>300 patients), possibly in the context of a prospective observational study. If the accuracy of the model is prospectively confirmed, a prospective interventional trial may be designed to identify a subset of patients that can be safely monitored without being isolated and/or the need of being testing for COVID-19.

**Summary points**

- This retrospective study explored the value of lung ultrasound as a predictor of a positive reverse transcriptase-polymerase chain reaction (RT-PCR) for severe acute respiratory syndrome coronavirus 2 in the emergency setting during the COVID-19 pandemic.
- A simple model based on lung ultrasound findings, aspartate transaminase (AST) levels and fever showed an overall accuracy of 91%.
- The proposed model showed an overall accuracy that was comparable to that of computed tomography (CT) findings.
- Prospective validation of the model is warranted.
